# Relationship Between Evidence Requirements, User Expectations, and Actual Experiences: Usability Evaluation of the Twazon Arabic Weight Loss App

**DOI:** 10.2196/humanfactors.9765

**Published:** 2018-04-17

**Authors:** Aroub Alnasser, Janet Kyle, Abdulrahman Alkhalifah, Debbi Marais

**Affiliations:** ^1^ Food Science and Nutrition Department College of Food and Agriculture Sciences King Saud University Riyadh Saudi Arabia; ^2^ Institute of Applied Health Sciences University of Aberdeen Aberdeen United Kingdom; ^3^ Warwick Medical School University of Warwick Coventry United Kingdom

**Keywords:** mHealth, weight loss, obesity, smartphones, mobile applications, Saudi Arabia, women's health

## Abstract

**Background:**

Saudi Arabia has faced a steady growth in the prevalence of obesity. The concurrent and ubiquitous use of mobile technology, such as smartphones and apps, provides an opportunity for the implementation of mHealth technology, a method for delivering behavioral interventions. Despite their effectiveness in promoting lifestyle and diet modification, culturally adapted weight loss apps and related interventions are lacking in Gulf Cooperation Council countries.

**Objective:**

The objective of our study was to identify the relationship between adherence to evidence-informed practices, potential user expectations, and actual user experiences in order to enhance the understanding of the overall usability of the Twazon Arabic weight loss app.

**Methods:**

In 2 previous studies, 39 Saudi women were recruited for focus group discussions and 240 Saudi women were recruited for an app-based weight loss intervention. Usability of the Twazon Arabic weight loss app was evaluated by analyzing the opinions and experiences of 26 participants who engaged with the Twazon app for 4 months; the System Usability Scale (SUS) and word clouds were used. The results were triangulated with potential user expectations obtained in the focus group discussion and with the findings from an Arabic app screening for evidence-informed practices.

**Results:**

The average reported SUS score was 69.3. The most favored features were the calorie counter, step counter, and physical activity calorie counter. The features in need of improvement were the social network, notifications, and the Twazon Saudi Food Database. Twazon users preferred and found useful 7 of the 13 evidence-informed weight loss practices that were integrated into the features of the app.

**Conclusions:**

Triangulation identified the most notable relationship to be the disparity between user experience and 2 of the evidence-informed practices, namely a minimum weight loss goal of 0.5 to 1 kg/week and social support; no relationship was found between user expectations and evidence-informed weight loss practices. The overall usability of the Twazon Arabic weight loss app ranged between high marginal and acceptable, indicating that some improvements to the app should be considered for implementation in future app-based weight loss interventions of this kind.

## Introduction

It is no longer news that obesity is a problem in Gulf Cooperation Council countries such as Saudi Arabia, affecting more women than men on average. A major driver of this is unhealthy behaviors such as physical inactivity, overeating, and unhealthful food choices [1]. Due to the severity of the epidemic, it is necessary to implement various treatment strategies and conduct interventions that are accessible to a larger population and effective over the long term. As a novel manner in which to deliver behavioral interventions that might be effective in lifestyle and diet modification, implementation of health-related technology, or rather mHealth, has been of emerging interest.

mHealth is a type of electronic health support that is defined as medical and public health practices that are promoted by mobile devices, such as smartphones, patient monitoring devices, personal digital assistants, and other wireless devices [2]. Commercial weight loss apps have been reported to be more engaging than those that are evidence-informed [3]; however, the quality of the information given by the commercial apps is often rated as low [4,5]. It follows that a more comprehensive user-centered design approach [6] that is based on evidence-informed practices, as well as user expectations and experiences, is vital to ensuring the efficacy of mHealth interventions.

Due to the widespread use and accessibility of mobile technology in Saudi Arabia [7], smartphone apps offer a substantial opportunity to support health behavior change and weight management. However, none that are evidence-informed and culturally adapted are available in the region. With the goal of implementing a 4-month weight loss intervention in Saudi Arabia (AA et al, unpublished data, 2017), the Twazon Arabic weight loss app [8] was developed based on the aforementioned factors in addition to behavior strategies [9], such as self-monitoring. To ensure the proper implementation of a complex intervention [10] involving a website or mobile app, *usability* —or how effectively, efficiently, and satisfactorily a user can interact with a user interface [11]—must be investigated.

The Twazon app was designed to be used autonomously by individuals (male or female) who have weight issues, but are otherwise healthy; it is not intended to be used as treatment in a health care system. The prevalence of overweight and obesity among Saudi women, and a scarcity of research done for this demographic, justify the need for a public health intervention to be carried out for women in this region. In this study, we aimed to identify the relationship between adherence to evidence-informed practices, potential user expectations, and actual user experiences in order to enhance the understanding of the overall usability of the Twazon weight loss app. A triangulation analysis revealed the relationship and tensions found between these aspects of the app’s components, and the results we report here reflect their compliance with the Twazon app.

## Methods

### Design Phase: Evidence Requirements and User Expectations

Weight loss apps in general have been found to be lacking in evidence-informed practices, and the majority that are available are commercial and in English. Due to a complete lack of a systematic reviews of weight loss apps in the region, the first step in designing Twazon [8] involved screening 65 Arabic weight loss apps for their adherence to evidence-informed practices [12] as recommended by various health authorities [13-15].

To further inform the development of the Twazon app, a qualitative study was conducted comprising 4 focus group discussions with the goal of determining potential users’ preferences and expectations in a weight loss app. A total of 39 Saudi women with overweight and obesity in Riyadh, Saudi Arabia [16] gave oral responses, which were transcribed and translated from Arabic into English by a certified bilingual translator. Discussions were thematically analyzed and categorized for each of the main topics, and specific quotations were identified to correlate with the theme in mind.

### Implementation Phase: App Development and Intervention

The result of the app screening and the focus group discussions was the selection of 13 individual evidence-informed practices, which were grouped as follows: weight assessment and goal setting, healthy diet, physical activity, self-monitoring, and social support (see Multimedia Appendix 1). The behavioral strategy of self-monitoring translated to features of the Twazon app that enable users to track their progress.

**Figure 1:**
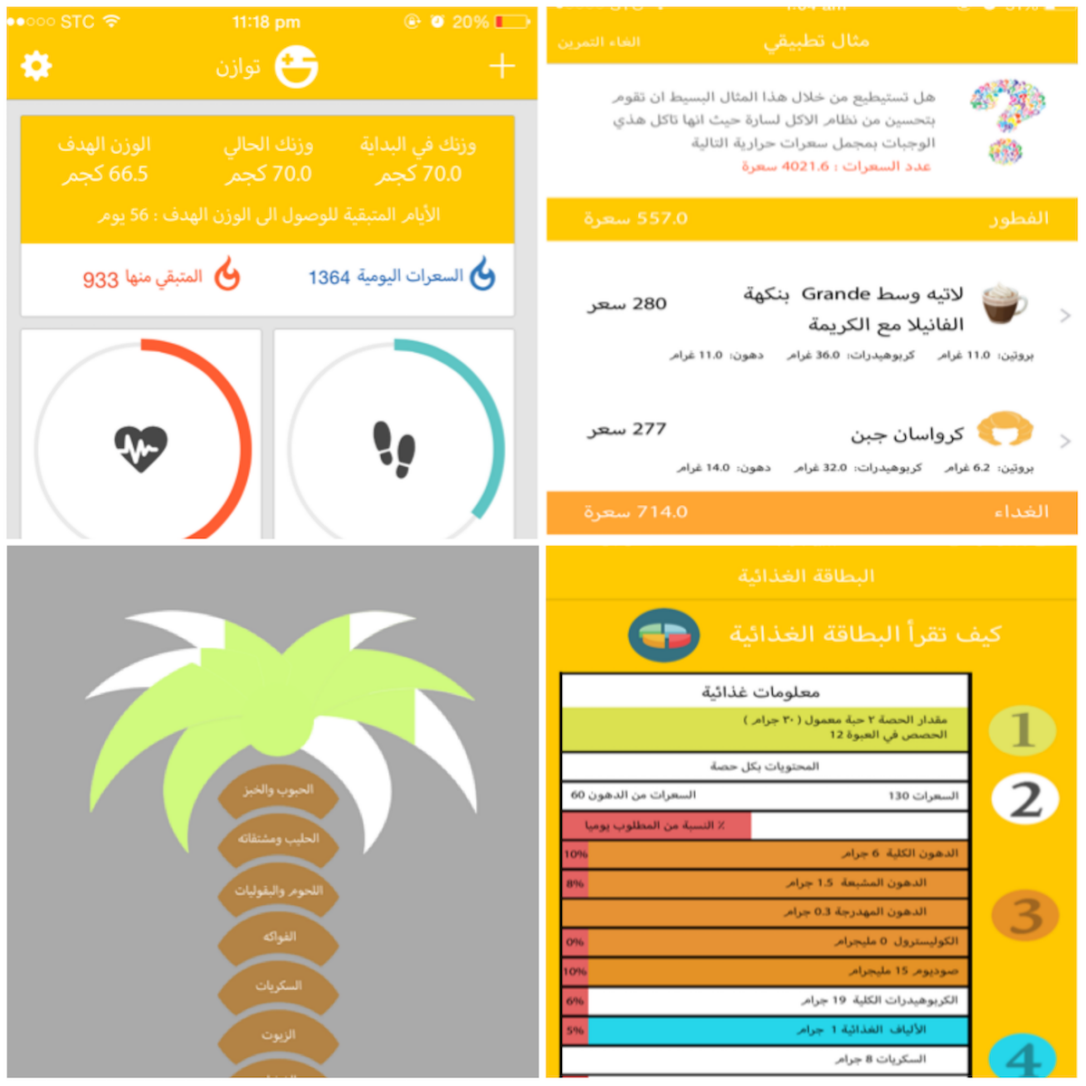
Twazon app interface (from top left, clockwise): Twazon dashboard, educational tool; food label tips, and healthy food palm.

The Twazon app requires a single log-in, must be connected to the Internet to function properly, and continues to work in the background. It is not a commercial app; it was developed and made freely available to the public through the iTunes (Apple Inc) and Google Play (Google LLC) stores. Daily physical activity, by activity and time spent doing it, is calculated with data from the user-updated physical activity journal and the integrated pedometer; daily water and energy intake are calculated based on user-updated input of consumption (Figure 1 shows the Twazon app interface).

The dashboard provides automatized, individually tailored, user-specific information regarding daily activity, consumption, and goal tracking, which is reset at the beginning of each day by an automatic algorithm. The food palm gives a personalized biweekly graphic display of the user’s healthy lifestyle self-assessment score, including physical activity tips when physical activity goals are not met. This feature is also designed to give instant feedback to users if they exceed their daily energy intake goal. The educational tool is used for menu planning, and the food label tips are used to understand the nutritional content of foods consumed. The Twazon app also offers social support, accessible at the bottom of the interface. This social network, which is restricted to users, encourages individuals to share personal health achievements with one another through the posting and liking of images and text; no other human contact or feedback from the developers is provided.

### Postintervention Phase: App Use and Usability

Engagement was based on app use, which was calculated by an automatic algorithm that grouped the participants according to the frequency of user input (AA et al, unpublished data, 2017). This was a necessary step in assessing usability in that only those participants who regularly updated their information could be considered.

**Figure 2:**
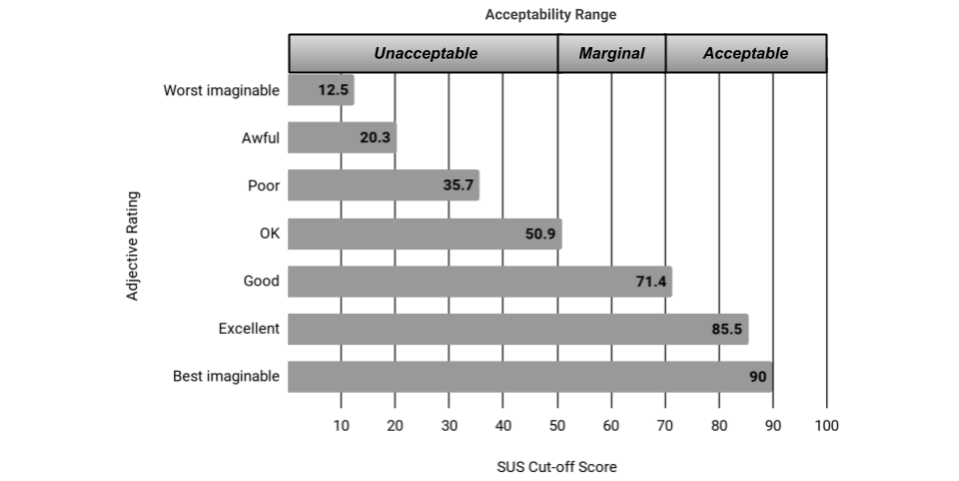
Grade rankings of System Usability Scale (SUS) scores. Adapted from Bangor et al [18,19].

Generally, usability testing conducted with 5 participants will identify at least 85% of usability problems [17]; in this study, a sample of 26 users was deemed to be more than sufficient. Participants were asked to assess the overall usability of the app during an individual interview at a predetermined location for 10 to 15 minutes. The overall usability score of the Twazon app was measured using the 10-question System Usability Scale (SUS), which generates a SUS score ranging from 0 to 100 that is associated with a 7-point adjective rating scale: worst imaginable (12.5), awful (20.3), poor (35.7), ok (50.9), good (71.4), excellent (85.5), or best imaginable (90.9) [18]. To determine what is an “acceptable” SUS score for a product to have, or rather whether a product requires more attention and continued improvement, the score is further classified by acceptability ranges—that is, not acceptable (0-50), low/high marginal (51-69), and acceptable (70 and above; Figure 2); higher product acceptability means fewer usability difficulties experienced by a user [19].

The SUS questions were ranked according to a 5-point Likert scale [20]. Each of the 10 questions had a score range set from 0 to 4. For responses 1, 3, 5, 7, and 9, the score was calculated by subtracting 1 from the scale value. For responses 2, 4, 6, 8, and 10, the score was calculated by subtracting the scale value from 5. The overall score was the total of the scores multiplied by 2.5 [18]. To identify which features of the app participants believed to be the most efficient and which could be improved upon, the participants were asked 2 open-ended questions that were added to the SUS questionnaire but analyzed separately: “What part of the app do you feel works the best?" and “Are there any parts of the app that you feel could be improved, and how?” The results obtained from the 2 additional questions were used to generate visual representations (word clouds) with free online software (Wordle [21]. Word clouds has predominantly been found in social and commercial settings; however, studies have shown that their use in analysis provides “a rapid and practical way to analyse textual data” and helps in “reducing the textual data without bias” [22].

This process allows the reader to quickly identify the most commonly used terms or responses in a given text as it entails illustrating a set of related tags or words in which frequency of word use is reflected visually through font size [23]; this represents the number of participants who gave a response, rather than the total number of responses, and is vital to eliminating the possibility of the same or similar comments being counted multiple times during individual interviews. The answers were sorted into 2 groups (app preferences and app improvements) and a word cloud was generated showing common themes for each question, for a total of 2 word clouds.

The collective results were prepared for analysis in a cross-comparative table using the 13 evidence-informed practice requirements. The information collected in the screening phase was used as a basis for identifying whether these linked to user expectations or experiences.

## Results

The Twazon weight loss app intervention was completed by 40 Saudi women with overweight or obesity over the course of 4 months; the rate of attrition was 83%. For the analysis that follows, only the data for the engaged participants (n=26) were used.

### System Usability Score (Twazon Intervention)

**Figure 3:**
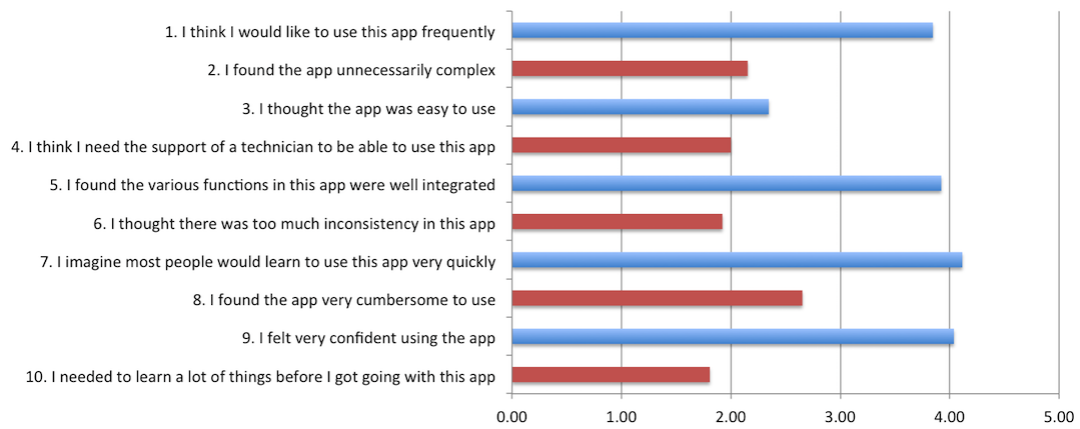
Mean System Usability Scale (SUS) scores corresponding to the 10 questions. Odd-numbered questions indicate a positive response, while even-numbered questions indicate a negative response. Higher numbers indicate increasing degrees of participant agreement.

The overall mean SUS score was 69.3 (SD 10.1), equating to an adjective rating of ok (average=50.9), which suggests that the participants found the app to be more than satisfactory. When compared with the averages for each adjective rating, however, this study’s scores were closer to a rating of good (average=71.4); this translates to an overall acceptability that ranges between high marginal and acceptable (see Figure 2). The highest-rated positive statements responses were numbers 7 (“I imagine most people would learn to use this app very quickly.”) and 9 (“I felt very confident using the app.”). The lowest-rated negative statements were numbers 10 (“I needed to learn a lot of things before I got going with this app.”) and 6 (“I thought there was too much inconsistency in this app.”); see Figure 3.

### Word Clouds (Twazon Intervention)

**Figure 4:**
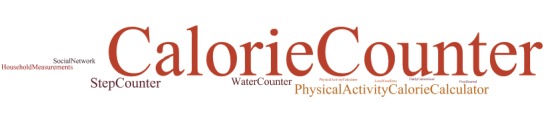
Word cloud representation of responses indicating the most preferred features.

**Figure 5:**
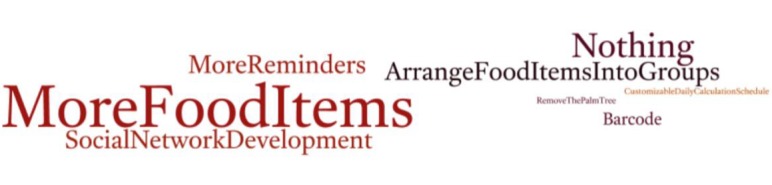
Word cloud representation of responses indicating features in need of improvement.

We generated 2 word clouds for the responses given for the 2 open-ended questions regarding the features that were most preferred (question 1) and those that were in need of improvement (question 2). The results for question 1 (Figure 4) showed that the most favored features of the app were the calorie counter, followed by the physical activity calorie calculator and the step counter. The water counter was the fourth most favored feature. The results for question 2 (Figure 5) showed that the primary suggested improvements were to have more food items, followed by change nothing, and then to add more reminders, arrange food items into groups, and social network development.

### Triangulation

The results of the screening indicated that Arabic weight loss apps had a very low adherence to evidence-informed practices (median=1); no apps had more than 6 evidence-informed practices, and only 9 apps had 4 to 6 integrated, which justified the need to develop an evidence-informed Arabic app. The focus group discussions then led to exploring potential users’ expectations of an ideal app. The results from those discussions indicated that the participants expected all 13 evidence-informed practices to be present in some feature of an ideal app, in addition to its being culturally adapted in terms of language, food, and exercise options (Table 1).

The results obtained from the SUS questionnaires and word clouds indicated that the Twazon users preferred and found useful more than half (7/13) of the evidence-informed practices that were integrated into the apps’ features. The participants reported that 2 of the 13 practices were insufficient (weight loss goal of 0.5-1 kg/week and social network), while they did not mention the remaining practices. A cross-comparative analysis (Table 1) highlighted the relationship between adherence to evidence-informed practices and overall usability of the Twazon weight loss app.

**Table 1:** Cross-comparative analysis of triangulation exploring potential users’ expectations of an ideal app. N/A: not applicable. NC: no comment.

Evidence-informed practices	Arabic weight loss apps (adherence; n=65), n (%)	Focus group discussion (expectations)	Twazon intervention (experiences)
1. Meal planning	25 (38)	Yes	Yes
2. Assessing your weight	17 (26)	Yes	NC
3. Regular physical activity	13 (20)	Yes	Yes
4. Maintaining calorie balance	10 (15)	Yes	Yes
5. Keeping a food diary	9 (14)	Yes	Yes
6. Portion control	0 (0)	Yes	NC
7. Eating a diet rich in fruits and vegetables	7 (11)	Yes	Yes
8. Tracking your weight	6 (9)	Yes	NC
9. Keeping a physical activity journal	6 (9)	Yes	Yes
10. Weight loss goal of 0.5-1 kg/week	5 (8)	Yes	No
11. Social support	2 (3)	Yes	No
12. Reading nutrition facts labels	2 (3)	Yes	NC
13. Water instead of soda/juice	7 (11)	Yes	Yes
Additional: culturally sensitive	N/A	Yes	NC
Additional: notifications	N/A	N/A	No

## Discussion

### Principal Findings

The Twazon app aimed to fill a gap in the research and development of evidence-informed Arabic weight loss apps [12] and interventions in order to find the optimum balance between evidence requirements and user needs. The participants’ experiences with the Twazon intervention provided insight into the features of the app that were the least interesting, the most effective, or in need of improvement. When considering the results from the triangulation analysis, a relationship emerged between what is perceived as the best or required practice from the evidence and what participants actually experienced and reported as being useful. Although the Arabic apps failed in general to meet requirements for all 13 evidence-informed practices, the women who took part in the focus group discussions [16] clearly communicated their expectation that all of them should be integrated into an ideal weight loss app.

Some of the evidence-informed practices, such as assessing one’s weight and tracking one’s weight, were not featured in the word clouds as being favored or in need improvement; this could be attributed to their essential nature in general weight loss programs and apps. The practice of eating a diet rich in fruits and vegetables and the practice of reading nutrition facts labels were also recommended by the app; neither was reported as favored or in need of improvement. Analysis of the SUS score for the question regarding the consumption of fruits and vegetables (AA et al, unpublished data, 2017), however, showed that the participants were successful in increasing their intake. This suggests that the app was effective in promoting this diet modification and practice.

Portion control was also recommended by the app, but the results from the word clouds gave no indication that this was either favored or in need of improvement. This could be attributed to a lack of typical serving sizes, which are found in other countries or in other databases. The development of the Twazon app included the creation of the Twazon Saudi Food Database (AA et al, unpublished data, 2017) with the goal of providing users with a detailed list of household measurements for local and international foods to help promote portion size awareness. However, the portion control feature was not mentioned by participants, implying a need for further investigation into the most effective manner in which it should be implemented.

The most commonly user-reported preferences and proposed improvements suggested that the users were more satisfied with the functions of the app (eg, counters) than with the content (eg, missing food item information).

### Primary Preferences

The results showed that the most favored features of the app were related to counters (Figure 4). The 4 evidence-informed practices that fulfill the reported preferences for most favored features are maintaining calorie balance (calorie counter), engaging in regular physical activity (step counter), keeping a physical activity and food journal (physical activity calorie calculator and calorie calculator), and drinking water instead of soda or juice (water counter). Our findings contrast with a recent qualitative study of 24 volunteers that suggested that counters are generally not preferred [24].

These results could be due to the fact that weight loss apps, and more specifically Arabic language apps that are culturally adapted, are relatively new to the region [25] and may be considered a novelty. A quote from one of the participants using the Twazon app illustrates this: “I was using an English weight loss app and against my better judgement I opted to eat pizza and burgers instead of kapsa or jarish so that I could count my calories with this app.” The act of counting calories may have been preferred in this study as a result of the participants’ interest in being able to log foods that they were familiar with due to their accessibility in the Twazon Saudi Food Database (AA et al, unpublished data, 2017). In future app development for the Gulf region, counters such as those found in the Twazon app could potentially be useful, as long as the practice remains novel.

### Primary Improvements

The primary suggested improvements were to have more food items, followed by have more reminders, arrange food items into groups, and social network development (Figure 5). The second most reported improvement was to change nothing; possible reasons for this were that the users were satisfied with the app, they found the app to be better than their SUS score suggested, or that they simply didn’t report accurately. The suggestions for improvements offer an opportunity to reevaluate the features and structure of the app, with the aim being to inform future app development.

Improvements were mentioned in regard to having access to more food items that are ideally arranged into specific groups to allow users to better log their daily consumption. The users’ adoption of evidence-informed practices, such as meal planning and portion control, may have been hindered by not being able to enter or find certain foods with ease. However, the participants’ inability to report their energy intake could be attributed to a falsely perceived lack of information. In some instances, participants were entering misspelled food items, causing duplicates, or were entering lengthy descriptions of dishes instead of simple keywords; this complicated the task and made logging foods more demanding.

Food data input challenges could be overcome with the addition of a barcode feature, which was one of the three least-reported suggested improvements (see Figure 5). One qualitative study [26] showed that a barcode feature should be considered, as it might improve users’ overall opinion of the quantity and types of food items available, enabling users to update their food intake with the ease of scanning food labels. Future apps might then consider expanding the Twazon Saudi Food Database to include more foods, integrate an autocorrect feature for spelling issues, and offer a barcode scanner to simplify food data input. Further investigation into these features and the user’s perception of them is needed to test their efficacy prior to carrying out an intervention.

The Twazon app provided users with three different types of notifications: (1) tailored tips based on unmet goals in food groups and physical activity, (2) general tips for foods to consume and foods to avoid, and (3) a reminder to enter weight and fill in the food palm tree assessment [8]; we gave them the option to choose how often (every 2 days, every 3 days, and every week) they received the first and second types of notifications, but the third type was automatically delivered every 2 weeks on completion of the required input. Although this was done to avoid overwhelming the participants, one study by Freyne et al [27] found that 3 notifications daily did not frustrate the users, exemplifying that an increase in notifications is not necessarily a hindrance. We suggest that more communicative contact should be considered in the development of future weight loss apps so as to encourage users to record as much as possible.

The evidence-informed practice of having a minimum weight loss goal of 0.5 to 1 kg/week perhaps identified the greatest relationship; many women reported losing interest in participating in the intervention due to not being satisfied with the aforementioned goal. This outcome could be explained by an aversion to goals perceived as being impossible or unsatisfying [28]. Several studies showed that participants with obesity are not motivated by an overall weight loss goal of 5% to 10%, as is recommended by health professionals, but rather a weight loss goal of 22% to 34% [29-31]. This failure to meet the expectations of patients with obesity suggests that there needs to be a smaller disparity between actual and expected outcomes. If this is achieved, then the probability of negative effects that are seemingly caused by unmet expectations can be lessened, and in turn more positive weight loss outcomes [32] can be achieved.

Evidence-informed weight loss programs have suggested that social networking could have a positive effect on weight loss outcomes; social media-based reports and sharing via social media sites such as Twitter are effective in weight loss interventions [33], as they can help motivate and empower participants to work harder toward their goals. In the Twazon app, we created an original and private social network that was accessible solely to the users of the app. However, the intervention participants reported the need for more social network development. Despite the remarkably high rate of social media use in the region, it seems that Saudi female participants were not as inclined as expected to share and interact in regard to their weight loss experience within the closed group.

Lack of engagement with the social media aspect of the app could be attributed to the participants not having direct support from family and friends, as is typical on most popular social media sites; the Twazon app was accessible exclusively to registrants of the app. However, one study [34] found no significant differences in a 6-month weight loss intervention between 3 different groups, which included a podcast plus Twitter group. Regardless, our results from the word clouds show that participants desired more social media development, suggesting that the use of social media sites as a tool to help promote weight loss and connect weight loss intervention participants should be considered and optimized in future app-based interventions.

Despite the integration of evidence-informed features into the Twazon app, challenges with retention still arose. To improve retention in future app interventions of this kind, modifications to the social networking feature and an increase in the amount of contact with the user is highly recommended. The reported user experiences also suggest that more consideration needs to be given to establishing weight loss goals that are not demotivating in order to facilitate more successful weight loss outcomes.

### Conclusion

Participants deemed the Twazon app to be of acceptable usability. The triangulation analysis revealed the greatest relationship to be the disparity between user experience and 2 of the evidence-informed practices, namely, a minimum weight loss goal of 0.5 to 1 kg/week and social support. In contrast, user expectations coincided with evidence-informed practices and therefore did not provide any relationship. Once the aforementioned improvements are made, it would be feasible for health care providers to recommend the use of Twazon in weight loss programs that involve behavioral modification strategies. Further in-depth exploration through qualitative study is also needed to better understand the relationship observed so as to appeal to the motivating factors that drive participants toward successful outcomes in their weight loss goals when using weight loss apps.

## Appendix

Multimedia Appendix 1 Tools in Twazon addressing evidence-informed weight loss practices.

## Abbreviations

SUS: System Usability Scale
