# Supplementary material for: Relationship Between Evidence Requirements, User Expectations, and Actual Experiences: Usability Evaluation of the Twazon Arabic Weight Loss App
Source: JMIR Hum Factors. 2018 Apr 17;5(2):e16. doi: 10.2196/humanfactors.9765 (PMC5930174; doi:10.2196/humanfactors.9765)
Supplement: Multimedia Appendix 1 [file humanfactors_v5i2e16_app1.pdf]

Multimedia Appendix 1. Tools in Twazon addressing evidence-informed weight loss practices.

**5 Groups of EIPs**	13 EIPs	Corresponding App Functionality
**Weight Assessment and Goal Setting**	➢ Assessing your weight<br>➢ Weight loss goal of 1-2 lb p/wk<br>➢ Tracking your weight	Assesses weight by calculating body mass index and waist circumference<br><br>Allows users to set their ideal weight, and sets a target date for achieving the weight loss goal<br><br>Calculates the number of calories needed daily based on their target weight<br><br>Recommends a decrease of at least 600 calories consumed per day in order to achieve weight loss goal
**Healthy Diet**	➢ Water instead of Soda or Juice<br>➢ Diet Rich in Fruits and Vegetables<br>➢ Meal Planning<br>➢ Reading Nutritional Facts Labels<br>➢ Portion Control	Recommends daily servings/portions of all foods and beverages, including 6 cups of water per day<br><br>Recommendations given according to healthy lifestyle self-assessment score<br><br>Provides a customized healthy food palm based on the user’s intake report<br><br>Recommends the reading of labels and describes how to properly read labels<br><br>Offers some suggestions for healthy food options in place of unhealthy food items.<br><br>Allows users to correct a poor meal/diet as an education tool for menu planning<br><br>Tips are sent if the intake/activity ratio is off-balance according to healthy lifestyle self-assessment score
**Physical Activity**	➢ Regular Physical Activity	Recommends a minimum of 30 minutes of physical activity three times a week and allows users to assess their physical activity every 2 weeks<br><br>Tips are sent if the amount of physical activity is low<br><br>Recommends taking at least 10,000 steps and provides a pedometer that tracks the daily number of steps
**Self Monitoring**	➢ Keep Physical Activity Journal<br>➢ Keep Food Diary<br>➢ Maintain Calorie Balance	Allows users to track their daily food (calories) and water intake, and number of servings per food group, every 2 weeks<br><br>Allows users to self-assess their physical activity, every 2 weeks<br><br>Provides a weight loss tracker that informs user of current weight loss toward their goal weight (kg)
**Social Support**	➢ Social Support	Provides an app-specific message board allowing users to privately share experiences, weight loss goals achieved, and photos with other users<br><br>Allows users access to social networking services such as Twitter
